# Inhibition of *Trichophyton rubrum* by 420-nm Intense Pulsed Light: *In Vitro* Activity and the Role of Nitric Oxide in Fungal Death

**DOI:** 10.3389/fphar.2019.01143

**Published:** 2019-10-03

**Authors:** Hao Huang, Meiling Huang, Wenyi Lv, Yong Hu, Ruihua Wang, Xiufen Zheng, Yuetang Ma, Chunmei Chen, Hongfeng Tang

**Affiliations:** Department of Dermatology, Shunde Hospital, Southern Medical University (The First People’s Hospital of Shunde Foshan), Foshan, China

**Keywords:** *Trichophyton rubrum*, 420-nm intense pulsed light, nitric oxide, asymmetric dimethylarginine, keratinase

## Abstract

*Trichophyton rubrum* is a common dermatophyte of the skin. The aim of this experiment was to explore the role of nitric oxide (NO) in the inhibition of *T. rubrum* growth induced by 420-nm intense pulsed light (IPL). This study found that nitric oxide synthase (NOS) and NO levels were increased, whereas asymmetric dimethylarginine (ADMA) level, keratinase activity, and fungal viability were decreased after IPL treatment compared with the control condition *in vitro*. Moreover, micromorphology was damaged by IPL treatment. Fungal viability was increased, and the damage to the fungal structure was reduced after pretreatment with an NOS inhibitor (L-NMMA) compared with IPL treatment alone. Compared with IPL alone, pretreatment with L-NMMA decreased NOS expression and NO level and increased keratinase activity. We found that 420-nm IPL treatment can inhibit the growth of *T. rubrum* by regulating NO *in vitro*.

## Introduction

Infectious diseases caused by fungi are collectively referred to as fungal diseases and are classified as superficial fungal diseases and deep fungal diseases according to the depth of invasion in the human body (Aly, 1994). Superficial fungal diseases have a high incidence and a long duration and easily relapse. In addition, the long-term use of antifungal drugs results in some damage to liver and kidney function, and these drugs are not recommended for use by elderly people, people with poor liver and kidney function or pregnant woman (Schafer-Korting, 2003; Peres et al., 2010 and Wang et al., 2014). Therefore, it is necessary to find safe, effective, simple, and easy new methods.

A potentially useful antifungal technique is phototherapy, as a new *in vivo* and *in vitro* application. Many studies have demonstrated that aminolevulinic acid photodynamic therapy (ALA-PDT), a long-pulse-width neodymium-doped yttrium aluminum garnet (Nd : YAG) laser (1,064 nm) therapy, could provide beneficial effects for the treatment of fungal disease in the clinic (Sotiriou et al., 2010). Additionally, studies revealed that 532- and 1,064-nm Q-switched Nd : YAG lasers and 280-nm light-emitting diodes (LEDs) significantly inhibited the growth of *T. rubrum in vitro* (Vural et al., 2008;Cronin et al., 2014).

The use of intense pulsed light (IPL) has extended into many fields. The advantage of this therapy is due to its wide range of emission wavelengths and good safety (Babilas et al., 2010). Four-hundred-and-twenty-nanometer IPL can kill bacteria and reduce inflammation, and it can be used to treat *Propionibacterium acnes* infection (Chang et al., 2007). Our previous study found that 420-nm IPL can inhibit fungal growth by inducing oxidative stress in fungi and causing fungal oxidative damage (Huang et al., 2017).

Nitric oxide (NO) is an important signaling compound in biological systems (Ignarro et al., 1990;Hardison, 1998). In the previous decade, fungi were discovered to synthesize NO (Vieira et al., 2009), mainly through the enzymatic oxidation of L-arginine by nitric oxide synthase (NOS) (Stuehr, 1999; Alderton et al., 2001). Studies have shown that NO can protect the mycelia of edible fungi from the oxidative damage induced by heat stress (Kong et al., 2012); moreover, clinical studies have also found that NO is an effective antifungal agent and has marked therapeutic effects on resistant fungal infections (Deppisch et al., 2016). It is speculated that the excessive accumulation of NO may result in the production of various reactive nitrogen species (RNS) and nitrosative stress conditions, inducing lipid membrane peroxidation, DNA damage, and the S-nitrosylation of sulfhydryl residues in fungi, and leading to delayed spore germination and even fungal apoptosis (Moncada et al., 1989;Calabrese et al., 2009). Baltazar et al. (Baltazar et al., 2013) found that using Toluidine Blue O (TBO) as a photosensitizer and LED (630 nm) as a light source to inhibit the growth of *Trichophyton rubrum in vitro* can upregulate NOS, thereby increasing nitrosative stress in fungal cells and leading to fungal injury or even death. This finding shows that NO plays an important role in the growth of fungi, and we also consider that phototherapy intervention regulates the level of NO in the fungus and plays a therapeutic role in fungal diseases. Therefore, we infer that IPL may also induce nitrification damage in fungi to inhibit fungal growth.

All eukaryotes can produce asymmetric dimethylarginine (ADMA) (Lakowski et al., 2013). Studies have shown that ADMA is an inhibitor of NOS that causes vasoconstriction and hypertension in humans, and its structure is similar to the structure of L-arginine, which is a substrate for NO synthesis. ADMA inhibits NOS activity and NO production, causing a series of biological effects that can mediate oxidative stress, inflammatory reactions, and other processes (Leiper and Nandi, 2011; Walter and Ron, 2011). ADMA is now considered to be a biologically active agent in cardiovascular diseases (CVDs) (Kielstein et al., 2004). Currently, little is known about the role of ADMA in fungi; a decrease in ADMA is only known to affect the growth of *Saccharomyces cerevisiae* upon heat stress, stationary phase growth, or other stress states (Lakowski et al., 2015). However, the role of ADMA in filamentous fungi has not yet been studied.

Keratinases have been speculated to be the most important dermatophyte virulence factor of fungi (Samdani et al., 1995). *Microsporum canis* with strong keratinase activity induced more pronounced clinical symptoms in infected guinea pigs than *M. canis* with low keratinase activity (Viani et al., 2001). Aprotinin, an inhibitor of serine protease (a kind of keratinase), has become an adjunct to natamycin and fluconazole (Biswas et al., 2001). Thus, the level of keratinase activity can be used to indirectly assess the strength of fungal pathogenicity. Our previous studies (Huang et al., 2017) have found that 420-nm IPL can affect fungal virulence by modulating reactive oxygen species (ROS) production, resulting in a cascade of oxidative stress within the fungus; however, whether 420-nm IPL can also affect fungal virulence by regulating nitrosative stress is unknown.

In this study, we investigated whether 420-nm IPL could inhibit fungal growth by causing nitrosative damage in fungi. As *T. rubrum* is one of the most common species of dermatophytes, accounting for 90% (Peres et al., 2010), we selected it as our experimental subject. This study aimed to examine changes in NO, NOS, ADMA, keratinase, and fungal morphology to explore the role of NO in the inhibition of *T. rubrum* growth induced by 420-nm IPL and to further explore a new method for the treatment of infectious fungal diseases.

## Methods

### Fungus Source

A clinical isolate of *T. rubrum* was provided by Sun Yat-Sen Memorial Hospital, Guangdong, China. Another *T. rubrum* strain, ATCC4438, was obtained from the American Type Culture Collection (ATCC).

### Preparation of Isolates

The following isolates of both strains were used in this study: (Aly, 1994) one aliquot (10 µl) of the fungal spore suspension (5 × 10^4^ cfu/ml) was pipetted onto the Sabouraud medium, (Schafer-Korting, 2003) and spore suspensions (5 × 10^4^ cfu/ml) in growth medium consisting of polypeptone (10 g) and glucose (40 g) in water (1,000 ml) were cultured in 96-well flat-bottomed microdilution plates (100 µl per well) before IPL irradiation (Huang et al., 2017).

### Light Source

An IPL device (Contour Profile, Sciton Company, USA) was used in this study with a cutoff excitation filter set at 420 nm. The setting for each pulse was 150 ms, and the temperature setting was 10°C. For *in vitro* fungal intervention, the energy used was 12 pulses at 12 J/cm^2^ according to our previous experiments (Huang et al., 2018).

### Fungal Activity Detection

To explore the role of NOS in IPL treatment, we selected L-NMMA, an NOS inhibitor, to pretreat the fungus for 2 h before IPL treatment, and we detected fungal viability by an MTT assay 6 h after IPL irradiation.

### Determination of NO Level

According to the above experimental results, we divided the cultures in liquid medium into a *T. rubrum* group, a *T. rubrum*+IPL group and a *T. rubrum*+L-NMMA (0.4 mM)+IPL group. We selected N^G^-monomethyl-l-arginine, monoacetate salt (L-NMMA) to pretreat the fungus for 2 h before IPL irradiation. The *T. rubrum* suspension of each group was incubated with 5 μmol/L 3-amino,4-aminomethyl-2’,7’-difluorescein diacetate (DAF-FM DA, Beyotime Biotechnology, Haimen, China), a fluorescent probe, at 28°C for 30 min and washed in phosphate-buffered saline (PBS) twice. The fluorescent signal intensity was analyzed using a flow cytometer (BD FACS Canto II) at an excitation wavelength of 495 nm and an emission wavelength of 515 nm.

### Determination of NOS Level

After IPL treatment, the *T. rubrum* suspension was incubated with 100 μl NOS detection buffer and 100 μl reaction solution (Beyotime Biotechnology, Haimen, China) at 28°C for 30 min and washed in PBS twice. The fluorescent signal intensity was analyzed using a flow cytometer (BD FACS Canto II) at an excitation wavelength of 495 nm and an emission wavelength of 515 nm.

### Determination of ADMA Level

We divided the fungus in liquid medium into a *T. rubrum* group and a *T. rubrum*+IPL group. The supernatant of the *T. rubrum* suspension of each group was incubated with 50 μl of sample and 50 μl of Detection A at 37°C for 60 min and washed in detergent three times. Then, we added 100 μl of Detection B, incubated the mixture at 37°C for 30 min, and washed the plate five times. Next, 90 μl of tetramethylbenzidine (TMB) substrate was added and incubated at 37°C for 15–25 min. Finally, we added 50 μl of stop solution and immediately measured the optical density of each well with a microplate reader (SpectraMax M5, Molecular Devices, USA) at a wavelength of 450 nm. All the steps referred to the manufacturer’s instructions in a commercially available assay kit [enzyme-linked immunosorbent assay (ELISA)] made by Cloud-Clone Corporation, USA. (Only strain ATCC4438 was used in the ADMA detection assay.)

### Determination of Fungal Keratinase Activity

We added 500 μl of spore suspension (5 × 10^4^ cfu/ml) to 50 ml soy peptone medium at 28°C for 10 days. We divided the fungus in liquid medium into a *T. rubrum* group, a *T. rubrum*+L-NMMA group, a *T. rubrum*+IPL group, and a *T. rubrum*+L-NMMA+IPL group. Fungal keratinase activity was detected by using keratin-azure (Sigma), a widely used commercial reagent for the determination of keratinase. A total of 3 ml of supernatant per group was incubated with 10 mg of keratin-azure for 72 h at 37°C in 2 ml of buffer (0.555 g of CaCl_2_ in 50 ml of Tris-HCl buffer at pH = 8.0). Keratinase activity was detected by measuring the optical density at a wavelength of 595 nm. (Only strain ATCC4438 was used in the keratinase activity detection assay.)

### Morphological Changes in *T. Rubrum* After 420-Nm IPL Irradiation

The colonies were cut with a sterile blade, and pieces of the *T. rubrum*+L-NMMA+IPL group were incubated in PBS with L-NMMA for 2 h before IPL treatment as described by Xing et al. (Xing et al., 2013). The pieces of fungus were irradiated with IPL, and after 24 h, the samples were washed twice and fixed with 2.5% glutaraldehyde for 48 h at 4°C. The samples were sent to an electron microscope chamber for air-drying and gold spraying. Then, the samples were observed, and pictures were taken with a Hitachi S-3000N Scanning Electron Microscope (Japan). The electron microscope settings and magnification were condition = Vacc = 20 kV, Mag = ×4.7–5.0k, and bar = 10 µm.

### Data Analysis

The data are represented as the mean of six independent experiments in MTT assays. The others are represented as the mean of three independent experiments. All data were expressed as the mean ± SD. Statistical analyses were performed using a paired-sample t test and one-way analysis of variance (ANOVA), followed by *post hoc* analysis using the least significant difference (LSD) test or Dunnett’s T3 test. A P value of <0.05 was considered statistically significant.

## Results

### Effect of IPL Treatment on the Activity of *T. Rubrum*

**Figure 1:**
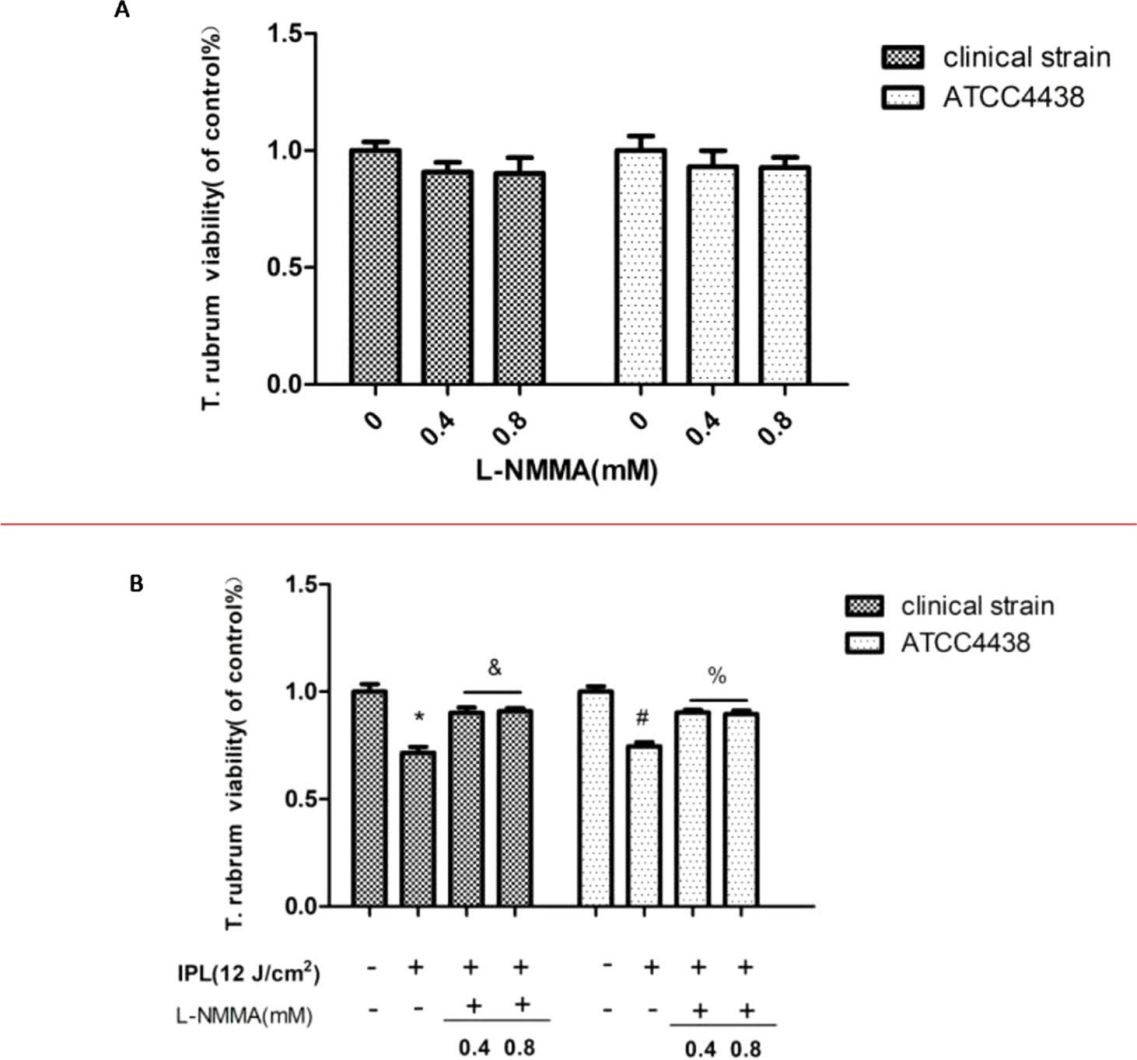
Effect of nitric oxide synthase (NOS) inhibitor (L-NMMA) on *Trichophyton rubrum*. **(A)** The effects of different L-NMMA concentrations. **(B)** The effect of L-NMMA on *T. rubrum* activity after intense pulsed light (IPL) treatment. (*P < 0.05 vs. untreated clinical strain; ^&^P < 0.05 vs. IPL-treated clinical strain; ^#^P < 0.05 vs. untreated ATCC4438; ^%^P < 0.05 vs. IPL-treated ATCC4438; all the control groups were considered 100%).

After the NOS inhibitor was added at different concentrations (0.4 and 0.8 mM), the fungal viability was not affected. According to these results, L-NMMA concentrations of 0.4 and 0.8 mM were selected for further analyses (**Figure 1A**). The NOS inhibitor group was incubated with different concentrations of L-NMMA (0.4 and 0.8 mM) for further IPL treatment (12 pulses at 12 J/cm^2^). The MTT assays showed that the inhibitor-treated group had significantly increased viability compared with the group treated with IPL irradiation alone (P < 0.05) (**Figure 1B**). However, there was no statistically significant difference between the L-NMMA–treated groups (0.4 and 0.8 mM). Therefore, the 0.4 mM concentration of L-NMMA was chosen for the following experiment.

### IPL Induces NO Generation in *T. Rubrum*

**Figure 2:**
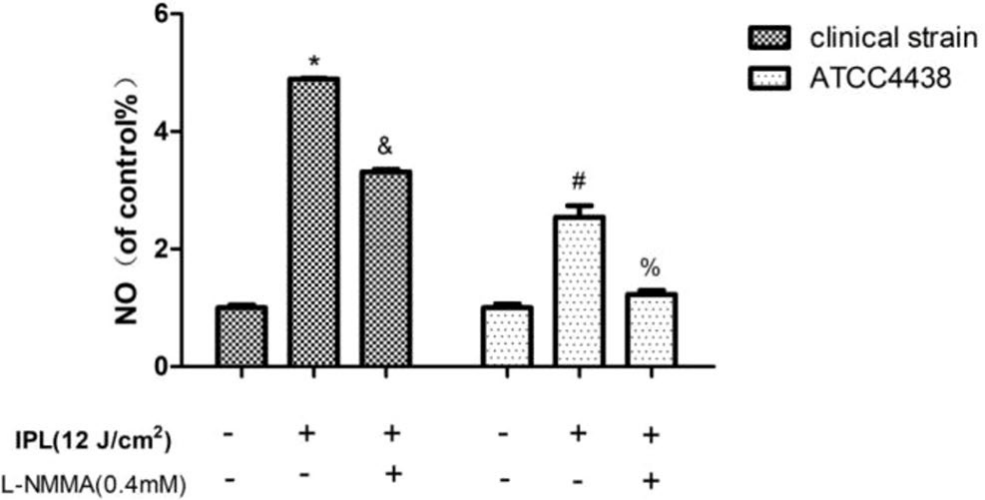
Changes in the intracellular nitric oxide (NO) levels of *T. rubrum*. (*P < 0.05 vs. untreated clinical strain; ^&^P < 0.05 vs. IPL-treated clinical strain; ^#^P < 0.05 vs. untreated ATCC4438; ^%^P < 0.05 vs. IPL-treated ATCC4438; all the control groups were considered 100%).

Compared to the control group, the *T. rubrum*+IPL group showed a significant increase (P < 0.05) in the level of NO. However, we observed that NO generation was markedly reduced in the *T. rubrum*+IPL+L-NMMA group compared to the *T. rubrum*+IPL group (P < 0.05) (**Figure 2**) (the exemplary dot plots in the **Supplementary Materials** of flow cytometry).

### NOS Expression in *T. Rubrum*

**Figure 3:**
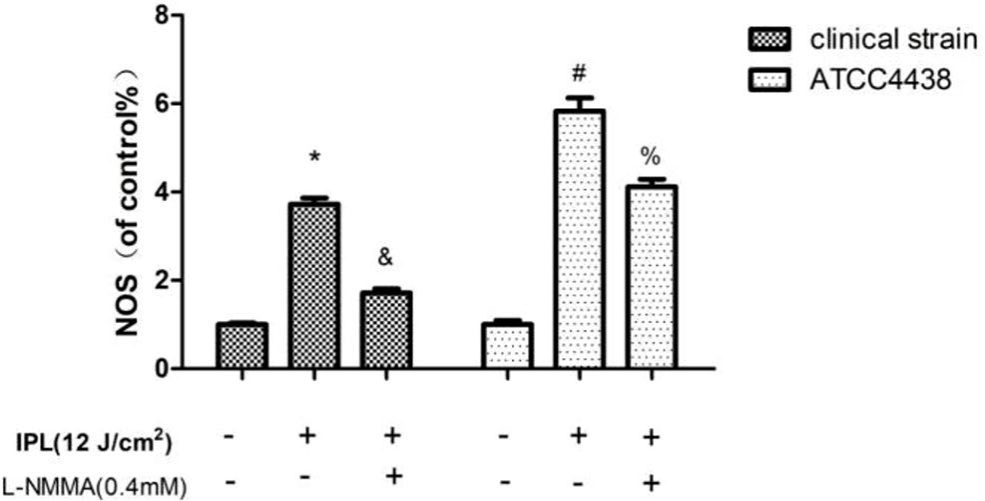
NOS expression in *T. rubrum*. (*P < 0.05 vs. untreated clinical strain; ^&^P < 0.05 vs. IPL-treated clinical strain; ^#^P < 0.05 vs. untreated ATCC4438; ^%^P < 0.05 vs. IPL-treated ATCC4438; all the control groups were considered 100%).

In our study, the results indicated that the expression of NOS was significantly upregulated in the *T. rubrum*+IPL group compared with the control group (P < 0.05). However, we observed that NOS expression was markedly reduced in the *T. rubrum*+IPL+L-NMMA group compared to the *T. rubrum*+IPL group (**Figure 3**) (the exemplary dot plots in the **Supplementary Materials** of flow cytometry).

### ADMA Expression in *T. Rubrum*

**Figure 4:**
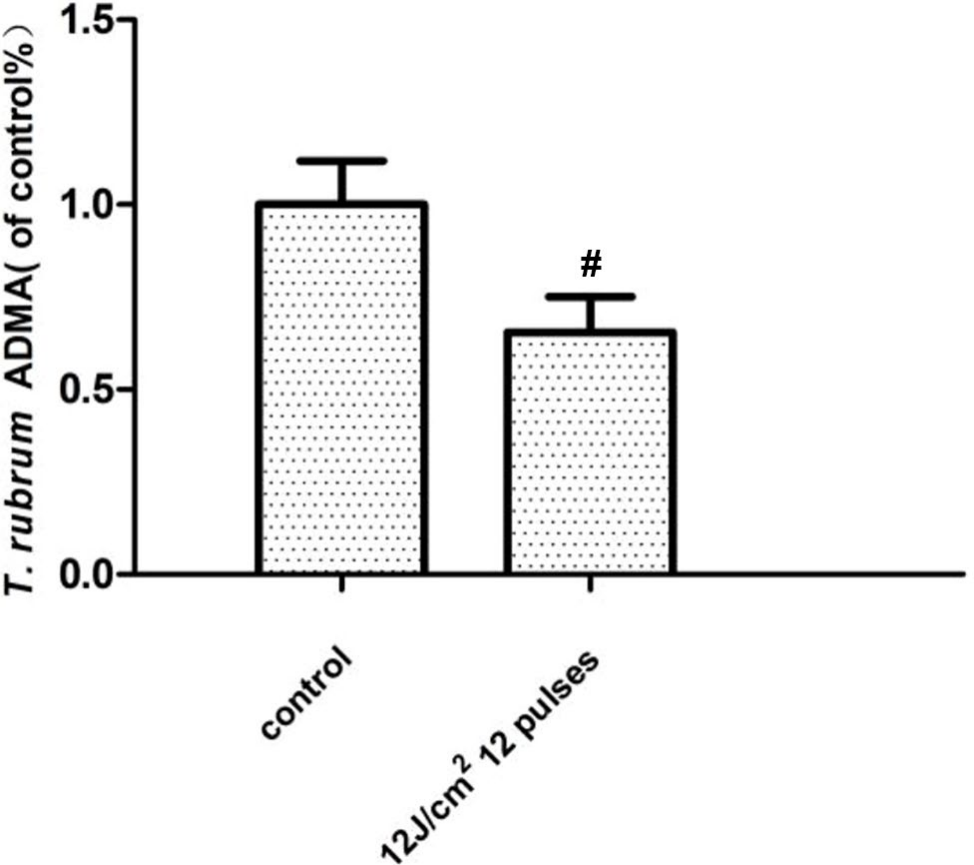
Changes in the asymmetric dimethylarginine (ADMA) expression of *T. rubrum*. (^#^P < 0.05 vs. untreated ATCC4438).

The results showed that the ADMA expression of the *T. rubrum* (ATCC4438)+IPL group was significantly downregulated compared with that of the *T. rubrum* (ATCC4438) group (P < 0.05) (**Figure 4**).

### Changes in Fungal Keratinase Activity

**Figure 5:**
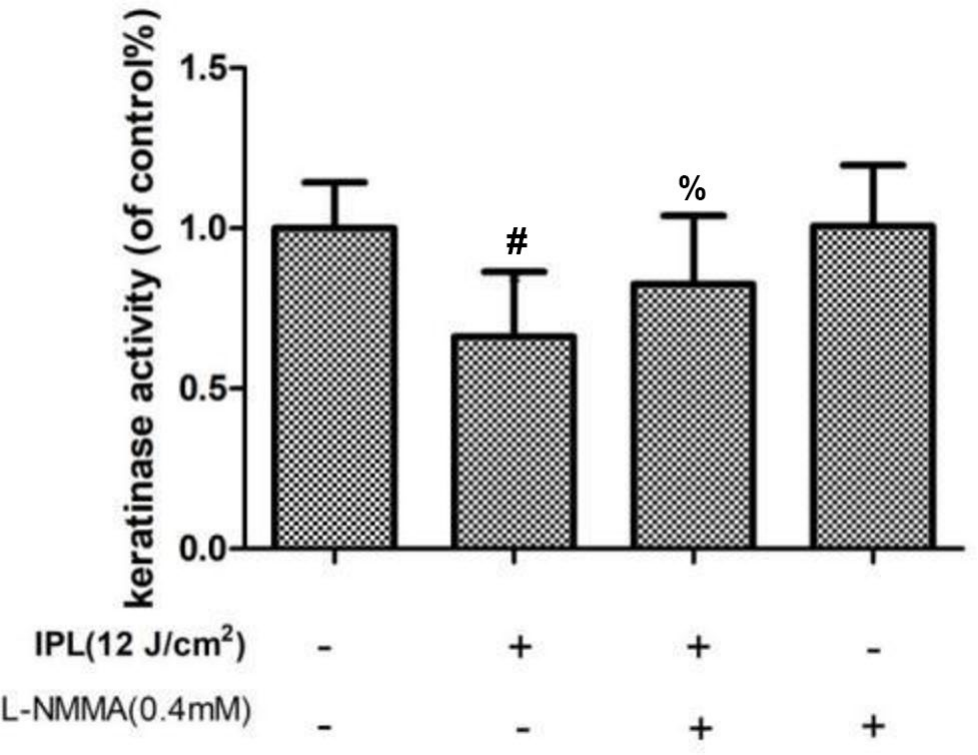
Effect of 24 h of IPL treatment on fungal keratinase activity. (^#^P < 0.05 vs. untreated ATCC4438; ^%^P < 0.05 vs. IPL-treated ATCC; all the control groups were considered 100%).

Compared with the control group, the group treated with IPL alone showed decreased keratinase activity, but the L-NMMA pretreatment group showed increased keratinase activity. And the activity of the group with L-NMMA alone had no statistically significant compared with the control group (P < 0.05) (**Figure 5**).

### Morphological Changes in *T. Rubrum* as Evidenced by SEM Examination

**Figure 6:**
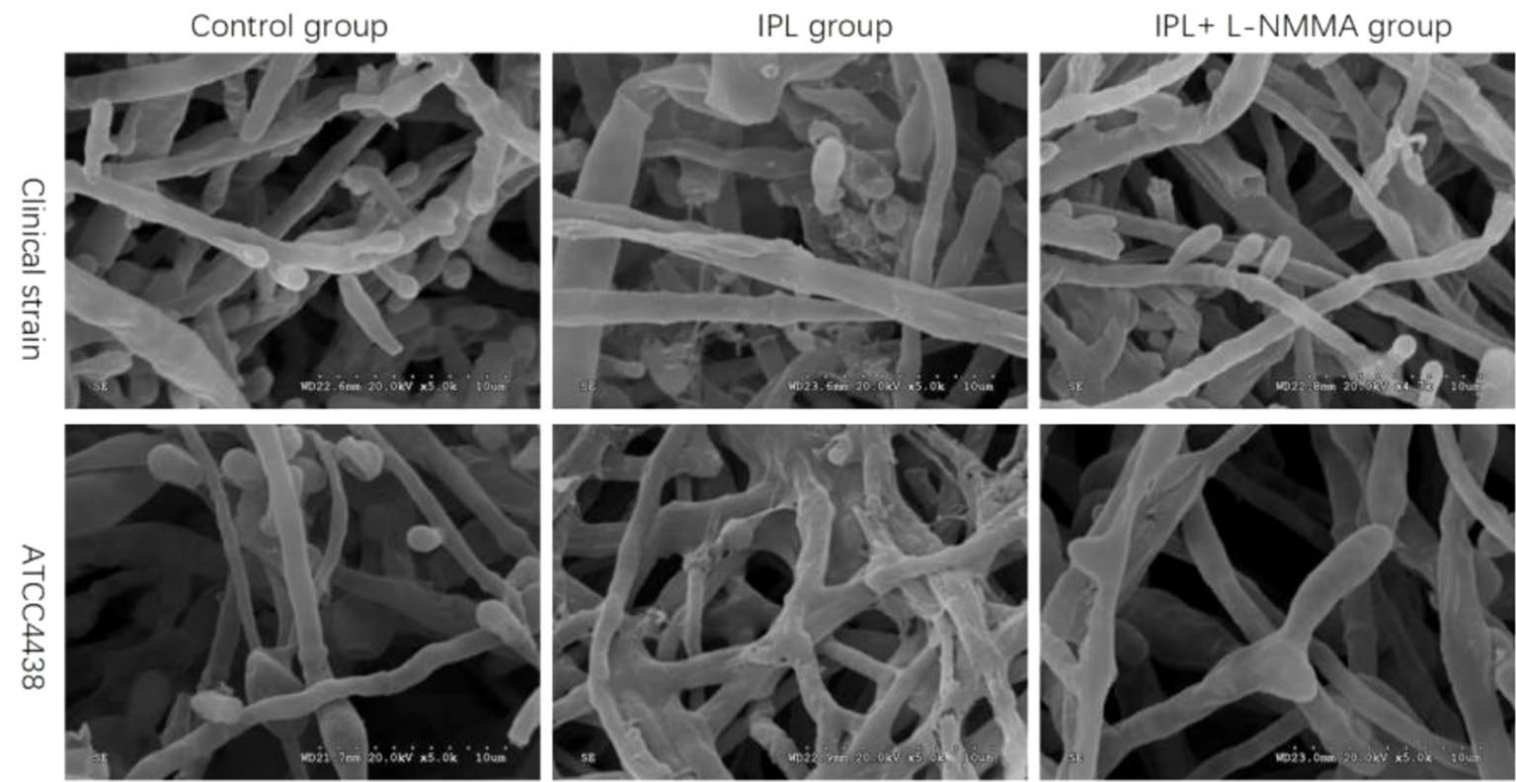
Scanning electron micrographs of *T. rubrum* with IPL treatment alone and L-NMMA pretreatment + IPL treatment for 24 h.

Under electron microscopy, the morphology of hyphae and spores in the control groups of both strains was normal; the surface was smooth and plump. The hyphae of the IPL-treated group were atrophic, distorted, shrunken, and irregular, with the emergence of many damaged and broken hyphae and surface deformation. However, compared with IPL treatment, L-NMMA pretreatment inhibited the morphological changes in hyphae and spores, which were uniformly thick, smooth, and plump, with almost no damage. Additionally, the fungus morphology was not significantly different in the *T. rubrum*+IPL+L-NMMA and the control groups (**Figure 6**).

## Discussion

In recent years, there have been many reports on the use of phototherapy in the treatment of fungal diseases. Yolanda Gilaberte et al. (Gilaberte et al., 2014) found that photodynamic therapy (PDT) effectively treats infectious fungal diseases such as sporotrichosis; Galvan Garcia HR (Galvan, 2014) found that the use of a 1,064-nm Q-switched Nd : YAG laser to treat onychomycosis (mainly caused by *T. rubrum*) is safe and effective. Currently, the US Food and Drug Administration (FDA) has approved a variety of lasers for the treatment of onychomycosis in clinical practice (Ledon et al., 2014).

IPL is different from monochromatic lasers in that it has a broad spectrum. Based on the selection of different filters, the device is mainly used for skin rejuvenation, hair removal, and freckle removal, and it also has a certain effect on acne (Hirsch and Shalita, 2003; Omi et al., 2004). To better explore the treatment mechanism of 420-nm IPL on fungal diseases, we selected *T. rubrum*, the most common dermatophyte, as an experimental subject for *in vitro* studies of 420-nm IPL irradiation.

NO is an important messenger molecule and effector molecule in fungal cells. It is closely related to the growth, morphogenesis, budding, propagation, and apoptosis of fungi (Chiranand et al., 2008). However, the half-life of NO is very short, and its biosynthesis mainly depends on the catalysis of NOS (Vieira et al., 2009). The expression of NOS is related to the amount of NO production. Therefore, detecting the change in NOS expression in *T. rubrum* after IPL treatment is a way to indirectly measure the change in NO. The synthesis of NO in fungi is mainly divided into oxidative and reductive synthesis. Under aerobic conditions, NOS in the cytoplasm catalyzes the production of NO by L-arginine, and the oxidative synthesis of NO has a dominant position; however, under hypoxic conditions, cytochrome-c oxidase (CcO) in mitochondria and nitrite reductase (NR) in the cytoplasm catalyze ${\text{NO}}_{2}^{-}$ to produce NO (Cassanova et al., 2005). Our experimental study found that after IPL intervention in *T. rubrum in vitro*, fungal activity was significantly decreased. scanning electron microscope (SEM) showed marked fungal destruction of the surface, accompanied by significantly increased NO levels, upregulated NOS, and decreased keratinase activity. However, compared with IPL treatment alone, the addition of an NOS inhibitor (L-NMMA) increased the activity of the fungi. Moreover, SEM showed that the surface of the fungus had almost no damage, while the NO level was decreased, the NOS expression was decreased, and the keratinase activity was increased in the group treated with L-NMMA and IPL compared with the group treated with IPL alone. According to our previous research results (Huang et al., 2017), *T. rubrum* produces a large amount of ROS under IPL intervention and forms an oxygen-rich state. Therefore, we can infer that the synthesis of NO in fungi occurs mainly through oxidative synthesis. IPL can induce NOS upregulation and catalyze NO production by L-arginine *via* NOS. NO is a dual-functional molecule. On the one hand, NO has important biological functions in fungi. NO can participate in the regulation of a variety of signaling pathways and in the transcription of certain genes (Chiranand et al., 2008); is involved in the regulation of cell oxidative stress, protein S-nitrosylation, and tyrosine nitrification (Tillmann et al., 2011), thus affecting the growth of fungi and morphogenesis; and plays a crucial role in maintaining the normal physiological activity of fungi. On the other hand, the excessive accumulation of NO causes nitrosative stress, which can cause damage to proteins, lipids, and DNA, resulting in fungal damage and apoptosis (Moncada et al., 1989; Calabrese et al., 2009). Therefore, combined with our experimental results, we speculate that IPL can increase the NO content in fungi by inducing NOS upregulation, causing fungal nitrosative injury or even death and decreasing the virulence of fungi.

Many methylarginines are targeted in yeast for protein synthesis, gene expression, and heat shock or nutritional deficiency responses. Studies (Herman, 2002; Verghese et al., 2012) have shown that yeast remain in the G1 phase of the cell cycle due to heat shock and stationary-phase status, and the observed ADMA concentration may also reflect the complete protein methylation pattern. The concentration of ADMA decreased by 11-fold during heat shock and the stationary phase compared to log-phase growth of wild-type *S. cerevisiae* (Lakowski et al., 2015). In human tissues and cells, ADMA is an endogenous competitive inhibitor of NOS that affects NO production and exerts a variety of biological effects. Studies have shown that ADMA is an independent risk factor for chronic kidney disease (CKD), CVD, and all-cause mortality (Fliser et al., 2005; Sydow et al., 2010). To study whether ADMA is also involved in the IPL-mediated inhibition of *T. rubrum* growth, we used 420-nm IPL to treat *T. rubrum*, not only to detect NOS expression and NO content but also to detect ADMA content. For the first time, we found that after the 420-nm IPL intervention, the activity of *T. rubrum* (ATCC4438) was decreased, which was accompanied by a decline in ADMA concentration, increased NOS expression, and increased levels of NO content. Combined with the role of ADMA in yeast and humans, we speculated that IPL can increase the expression of NOS by decreasing the content of ADMA, leading to an increase in the synthesis of NO, which causes nitrosative damage in fungi, thus inhibiting the growth of *T. rubrum*. We believe this is one of the important mechanisms of the IPL-mediated inhibition of fungal growth. However, the mechanism underlying the effect of IPL on ADMA content requires further study.

In summary, this study found that 420-nm IPL can significantly inhibit the growth of *T. rubrum in vitro*. Although the mechanism of this effect is not completely understood, the production of excessive NO and the induction of a series of reactions may play an important role. Based on our study, we speculate that this cascade of responses is caused by an increase in NOS induced by ADMA depletion, which may also be one of the targets of IPL on *T. rubrum*. In addition, combined with our previous experiments (Huang et al., 2017), after the 420-nm IPL intervention against the fungus, nitrosative stress and oxidative stress simultaneously existed. Additionally, Shen et al. (Shen et al., 2016) also found that ROS can participate in the thymol-induced death of *Aspergillus flavus* by inducing NO production. This result shows that there may be a certain relationship between ROS and NO. Therefore, we need to further explore the relationship between ROS and NO in the 420-nm IPL intervention of fungi to better understand the mechanism of the 420-nm IPL-induced inhibition of fungi. Based on our findings, 420-nm IPL may be a new potential method to treat fungal diseases.

## Data Availability Statement

The datasets generated for this study are available on request to the corresponding author.

## Author Contributions

HT was responsible for the experimental design. HH, XZ, YH, RW, YM, CC, WL, and MH were responsible for the experimental operation. MH, HH, and HT were responsible for writing the article.

## Funding

This research was supported by the Traditional Chinese Medicine Bureau of Guangdong Province (No. 20191317), Foshan Science and Technology Burea (FS0AA-KJ218-1301-0013), Clinical ResearchStart Program of Southern Medical University by High-level University Construction Funding of Guangdong Provincial Department of Education (No. PY2018N114), Scientific ResearchStart Plan of Shunde Hospital, Southern Medical University (No.SRSP2018014), Foshan Shunde Talent Development Service Center (Hongfeng Tang Expert Studio) and Foshan Medical Backbone Talents.

## Conflict of Interest

The authors declare that the research was conducted in the absence of any commercial or financial relationships that could be construed as a potential conflict of interest.
