# Supplementary material for: Inhibition of Trichophyton rubrum by 420-nm Intense Pulsed Light: In Vitro Activity and the Role of Nitric Oxide in Fungal Death
Source: Front Pharmacol. 2019 Oct 3;10:1143. doi: 10.3389/fphar.2019.01143 (PMC6785631; doi:10.3389/fphar.2019.01143)

1. NO: Fig a is the exemplary dot plots of control group (clinical strain) of flow cytometry. Fig b is the exemplary dot plots of IPL group (clinical strain) of flow cytometry. Fig c is the exemplary dot plots of IPL+L-NMMA group (clinical strain)of flow cytometry. From each sample, 10,000 events were collected. The three gates of flow cytometry were set by engineer of BD company. P1 is all fungal groups, P2 is a negative group(it was not added fluorescent probe) based on blanks, and P3 is a target fungal group (relative fluorescence intensity of expression).

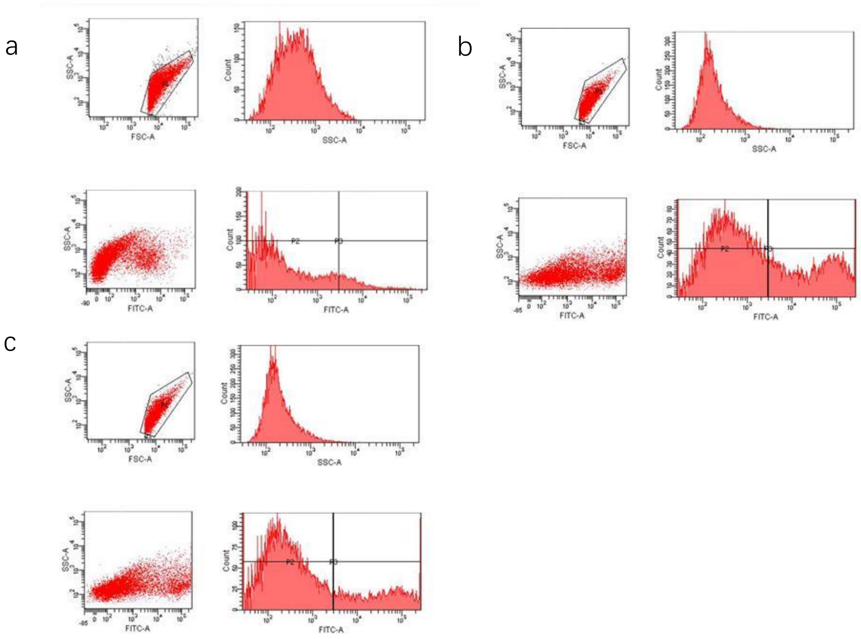

2. NOS: Fig a is the exemplary dot plots of control group of flow cytometry. Fig b is the exemplary dot plots of IPL group of flow cytometry. Fig c is the exemplary dot plots of IPL+L-NMMA group of flow cytometry. From each sample, 10,000 events were collected. The three gates of flow cytometry were set by engineer of BD company. P1 is all fungal groups, P2 is a negative group(it was not added fluorescent probe) based on blanks, and P3 is a target fungal group (relative fluorescence intensity of expression).

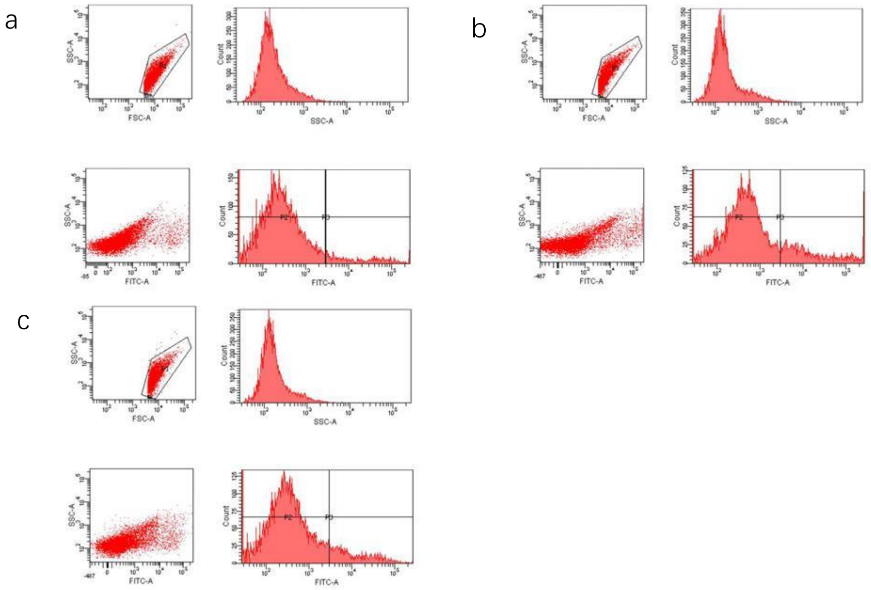

Supplement: Supplementary file 1 [file DataSheet_1.pdf]
